# N-3 Polyunsatured Fatty Acids in Menopausal Transition: A Systematic Review of Depressive and Cognitive Disorders with Accompanying Vasomotor Symptoms

**DOI:** 10.3390/ijms19071849

**Published:** 2018-06-23

**Authors:** Valentina Ciappolino, Alessandra Mazzocchi, Paolo Enrico, Marie-Louise Syrén, Giuseppe Delvecchio, Carlo Agostoni, Paolo Brambilla

**Affiliations:** 1 Department of Neurosciences and Mental Health, Fondazione IRCCS Ca’ Granda Ospedale Maggiore Policlinico, University of Milan, 20122 Milan, Italy; paolo.enrico@unimi.it (P.E.); paolo.brambilla1@unimi.it (P.B.); 2 Pediatric Intermediate Care Unit, Fondazione IRCCS Ca’ Granda-Ospedale Maggiore Policlinico, 20122 Milan, Italy; alessandra.mazzocchi@unimi.it (A.M.); eva.syren@unimi.it (M.-L.S.); carlo.agostoni@unimi.it (C.A.); 3 Department of Clinical Sciences and Community Health, University of Milan, 20122 Milan, Italy; 4 Department of Pathophysiology and Transplantation, University of Milan, 20122 Milan, Italy; g.delvecchio@hotmail.it; 5 SIGENP (Italian Society of Pediatric Gastroenterology, Hepatology, and Nutrition), via Libero Temolo 4 (Torre U8), 20126 Milan, Italy; 6 Department of Psychiatry and Behavioural Neurosciences, University of Texas at Houston, Houston, TX 77030, USA

**Keywords:** menopausal transition, perimenopause, omega-3, n-3 LCPUFAs, EPA, DHA, hot flashes, depression, cognition, affective disorders

## Abstract

Depression is one of the most important health problems worldwide. Women are 2.5 times more likely to experience major depression than men. Evidence suggests that some women might experience an increased risk for developing depression during “windows of vulnerability”, i.e., when exposed to intense hormone fluctuations, such as the menopause transition. Indeed, this period is associated with different symptoms, including vasomotor, depressive, and cognitive symptoms, which have all been shown to worsen as women approach menopause. Even though hormonal therapy represents the most effective treatment, side effects have been reported by several studies. Therefore, an increased number of women might prefer the use of alternative medicine for treating menopausal symptoms. N-3 long-chain polyunsaturated fatty acids (n-3 LCPUFAs) are included among these alternative treatments. We here provide a review of studies investigating the effects of n-3 LCPUFAs on hot flashes and depressive and cognitive disorders in menopausal women. The reported results are scattered and heterogeneous. In conclusion, a beneficial role of n-3 LCPUFAs in hot flashes, and depressive and cognitive symptoms related to menopausal transition is still far from conclusive.

## 1. Introduction

Depression is a chronic mental disorder with several relapses or recurrences, that currently affects 350 million people worldwide [1]. Despite the increasing number of antidepressant drugs, current treatment for depression will be effective for only 60% of patients [2]. For these reasons is important to develop novel therapeutic strategies to treat depressed patients.

Depression is much more common among women than men. Epidemiological data have showed that women are 2.5 times more likely to suffer from major depression than men [3,4], with some women appearing to be at higher risk during menopausal transition [5,6].

The terms perimenopause or menopausal transition, as defined by the latest consensus criteria for staging of reproductive aging (STRAW + 10), cover the transition from the reproductive age through to menopause and it is based on self-reported bleeding patterns. Perimenopause is characterized by three steps: early menopausal transition (−2), persistent cycle irregularity, considered as ≥7 days difference in length of consecutive cycles at least twice over the prior 10 cycles; late menopausal transition (−1), an interval of amenorrhea of ≥60 days in the prior 12 months; early post menopause (+1a), the first year following the final menstrual period (FMP) [7]. Indeed STRAW + 10 split out post menopause in two subgroups: early post menopause (i.e., the first six years following the FMP) and late post menopause (i.e., remaining lifespan) [7].

During the menopausal transition period, ovarian follicular functions reduce and subsequently the levels of estrogens (estradiol and estrone) and progesterone first become fluctuating and finally diminish, whereas levels of follicle stimulating hormone (FSH) increase [8,9]. These hormonal fluctuations lead to menstrual cycle irregularity [9,10], vasomotor instability [11], and cognitive [12] and affective changes [13,14,15].

Among the most invalidating symptoms of menopausal transition, there are hot flashes, depressive, and cognitive symptoms [16]. Hot flashes affect up to 80% of women during the menopausal transition and may last many years, ultimately compromising the quality of life [17].

Moreover, during the menopausal transition, the risk of onset of major depressive disorders (MDD) increases [18,19] and mood symptoms arise, becoming persistent in 15% to 18% of perimenopausal women, compared with 8% to 12% of premenopausal women [20].

Depression and hot flashes commonly co-occur during the menopausal transition, and women with hot flashes are at increased risk for MDD [21]. Therefore, many data showed that perimenopause is not only linked to a higher risk of depression but also to an impairment of cognitive functions [12,22,23,24,25]. Indeed, estrogens play an important role in promoting neuronal growth and survival [26], and it acts especially on the prefrontal cortex [27,28,29,30,31]; as well, as it exerts an effect on the cholinergic system, which is involved in cognitive functioning, especially memory [10,32].

Estrogens are also involved in the neurotransmitter systems implicated in the pathophysiology of depression, acting as serotonergic agonists/modulators by increasing receptor binding sites, synthesis and uptake both in animal models [33] and in post-menopausal women [34]. For these reasons, women can improve their perimenopausal–related depression as well as depressive symptomatology using estrogen therapy (ET) [35,36,37,38], which exerts a higher antidepressant effect in association with selective serotonin reuptake inhibitor (SSRI) treatment [39].

Furthermore, several women in menopausal transition show an impairment of cognitive functioning (e.g., forgetfulness and concentration difficulties), despite the weakness of the evidence exploring this aspect [40].

Although substitutive hormonal therapy is considered the first line treatment for mood and vasomotor symptoms menopause-related, a larger number of women tend to prefer alternative therapies, particularly herbal supplements (e.g., isoflavones and black cohosh), or treatment with selective serotonergic reuptake inhibitors (SSRIs), because of their reduced risks for health [17].

Nevertheless, most of these alternative medicines still remain understudied in the treatment of MDD and menopausal symptoms.

Among these supplements, n-3 long-chain polyunsatured fatty acids (n-3 LCPUFAs) are one of the most employed alternative therapies and are associated with other health benefits [41].

### Biologic Plausibility

The use of n-3 LCPUFAs in the menopausal period is derived from evidence showing that estrogens stimulate, while testosterone inhibits, the conversion of essential fatty acids into their longer chain metabolites, such as the case with α-linolenic acid conversion into docosahexaenoic acid (DHA) [42].

Omega-3 polyunsaturated fatty acid supplements mainly include gamma-linolenic acid (GLA), eicosapentaenoic acid (EPA) and DHA, both derived from the precursor essential fatty acid (EFA) alpha-linolenic acid (ALA), which are used for the treatment of several diseases (e.g., autoimmune and cardiovascular diseases, psychiatric and cognitive disorders) because of their involvement in numerous physiological functions, such as the composition of cell membrane structure and several cell functions and responses [43].

The enzymatic metabolic byproducts of LCPUFAs are called eicosanoids and include prostaglandins, thromboxanes, and leukotrienes. In particular, EPA-derived eicosanoids and products from DHA (called docosanoids) may exert anti-inflammatory effects, therefore influencing the final outcome of reactive processes [44].

Since n-3 LCPUFAs are incorporated into membrane phospholipids, the incorporation of DHA takes place at a uniquely high level in the central nervous system, where phospholipids dominate within the fat matrix [45]. A high-rate accumulation takes place in the brain starting in the brain growth spurt during the intrauterine and neonatal period up to two years of age, and these high levels are maintained throughout life [46], thus suggesting a continuous interaction with endogenous and dietary pools through the whole life-span.

Accumulating data of increasing n-3 LCPUFAs use in psychiatry has a primary biological basis in their neuroprotective properties, possibly producing modifications at the synaptic level [47]. Accordingly, n-3 LCPUFAs regulate brain cell signaling, including monoamine metabolism, and are involved in the modification of receptor properties, or the activation of signal transduction by receptors [48,49,50,51], at the basis of certain psychiatric diseases [52,53]. Human and animal studies exploring the mechanism of action of n-3 LCPUFAs suggest the involvement of these supplements in the modulation of serotonergic and dopaminergic neurotransmission [54,55,56].

The purpose of the present review is to synthesize the existing studies of the efficacy and safety exerted by n-3 LCPUFAs supplementation in hot flashes, depressive, and cognitive symptoms occurring during menopausal transition. Based on the abovementioned evidence it seems that n-3 LCPUFAs might be useful either alone or as coadjuvant therapy for these disabling perimenopausal symptoms.

## 2. Results

### 2.1. N-3 LCPUFAs in Menopausal Depressive and Cognitive Symptoms

Several epidemiological and observational studies suggest that a greater dietary intake of fish or a n-3 LCPUFAs supplementation is related to a general reduced risk of developing depressive disorders or fewer depressive symptoms [57,58]. Indeed, n-3 LCPUFAs exert their effects on cell membrane fluidity [59] and impact on important neurophysiological pathways, mainly serotonin and dopamine transmission, and their effects could be considered similar to those of selective serotonin reuptake inhibitors (SSRIs) [60]. For these reasons n-3 LCPUFAs have been implicated in the aetiology and in the treatment of different psychiatric disorders, in particular in MDD. Available data so far suggest that purified EPA or EPA-enriched, rather than purified DHA or DHA-enriched supplements, alone or as add-on therapy, are more beneficial in the treatment of MDD [61].

Furthermore, neuroimaging studies suggest that n-3 LCPUFAs promote cortical white matter microstructural integrity [62], and a recent study found that the supplementation of n-3 LCPUFAs increased white matter microstructural integrity and decreased depressive symptom severity in MDD patients [63].

It is of note that many incidence [64] and cross-sectional [58,65,66] studies with no fewer than 1300 participants have demonstrated an inverse correlation of n-3 LCPUFAs or fish with depressive symptoms or disorders in women but not in men. This gender-specific association could be explained by the estrogen–associated effects of n-3 LCPUFAs [67].

Specifically, menopausal depression show associations with previous Premenstrual Syndrome (PMS) or postpartum depression; suffering of menopause side effects such as hot flashes, night sweats, and insomnia; stress; weight gain; and low socio-economic level [68]. On the other hand, depressed menopausal women are also exposed to a higher risk of developing osteoporosis, bone loss [69], and cardiovascular disorders [70].

To clarify this issue, we carried out a review of all studies exploring the impact of n-3 LCPUFAs on depression during menopausal transition (Table 1).

The first randomized control trial (RCT) study was conducted by Lucas et al. in 2009 [71] on a sample of 120 postmenopausal women (aged 40–55 years old) with moderate-to-severe psychological distress as measured by the Psychological General Well-Being (PGWB) Schedule, and with depressive symptoms measured by the 20-item Hopkins Symptom Checklist Depression Scale (HSCL-D-20) and by the 21-item Hamilton Depression Rating Scale (HAM-D-21), randomly assigned to receive EPA (1.05 g/day) and DHA (0.25 g/day) or placebo for eight weeks.

At baseline only, a minority of depressed women (24%) satisfied the criteria for a major depressive episode (MDE). After eight weeks, outcomes improved in both groups, without significant differences between groups. Stratification analyses for MDE diagnosis showed that 8-week changes in psychological distress and depressive scales improved significantly only in women with psychological distress without MDE, but not in the MDE group. By contrast, Freeman at al. (2011) conducted an open label study exploring the efficacy of n-3 LCPUFAs (eicosapentaenoic acid and docosahexaenoic acid, 2 g/day) for MDE associated with the menopausal transition. Twenty menopausal women (aged 44–50 years old) received eight weeks of treatment and their depressive symptoms were measured by the Montgomery-Asberg Depression Rating Scale (MADRS). The authors showed that n-3 LCPUFAs had a significant antidepressant effect according to a decreased MADRS scores. Interestingly, responders had significantly lower pre-treatment docosahexaenoic acid levels than no responders. Despite the small sample size and the lack of a placebo control group, this can be considered a positive study that support the use of n-3 LCPUFAs for MDE during the menopausal transition [72].

In contrast, Cohen et al. (2014), in a larger multi-center RCT study, failed to demonstrate that n-3 LCPUFAs improve mood in menopausal women compared to placebo. The authors administrated 1.8 g/day of n-3 LCPUFAs supplementation (3 pills/day each containing: 425 mg of EPA, 100 mg DHA, and 90 mg of other omega-3s) or a placebo to a sample of 375 menopausal women (aged 40–62 years old) for 12 weeks. Primary outcomes were vasomotor symptoms (VMS) frequency. Secondary outcomes also included depressive symptoms as measured by the Physician’s Health Questionnaire-8 (PMQ-8). However, in this sample only a very small proportion of women (8%) had substantial depressive symptoms (PHQ-8 > 9), and therefore it was difficult to demonstrate the effect of n-3 LCPUFAs on mood [73].

Finally, we found only one RCT study exploring the combined effect of n-3 LCPUFAs and SSRI (citalopram) for the treatment of 60 postmenopausal depressed women (aged 45–65 years old) [74]. This is a triple-blind randomized controlled trial where the control group received 20 mg citalopram plus a placebo, whereas the intervention group received 20 mg citalopram and 1 g of n-3 LCPUFAs for a week. Beck’s Depression Inventory (BDI) was administered at baseline, and at the end of the first, second, and fourth weeks. A decreasing trend was observed in the mean depression scores of the intervention group during the study, with mean depression scores of the intervention group being significantly lower than the control group either two weeks or four weeks after the treatments. Therefore, these results suggest that n-3 LCPUFAs can reduce the severity of depression in post-menopausal women.

In conclusion, taken all together, these studies are not sufficient and powered enough to suggest whether n-3 LCPUFAs, alone or as add-on therapy, are beneficial for the treatment of MDE. However, better-designed and larger population-based studies are needed to clarify whether n-3 LCPUFAs are effective alone or in a synergistic way, combined with hormone therapy (HT) or SSRI, in particular at specific doses.

With regards to the linkage between n-3 LCPUFAs and cognitive functions in humans, several observational studies described a potential protective role of DHA in age-related cognitive decline [76,77]. Based on these evidences, it has been reported a positive effect of DHA supplementation on some cognitive outcome measures, such as Cambridge Neuropsychological Test Automated Battery (CANTAB) Paired Associate Learning (PAL) and the cognitive portion of the Alzheimer’s Disease Assessment Scale (ADAS-cog), in both healthy subjects, [78] and in patients with mild cognitive impairment [79] or Alzheimer’s disease [80].

Despite great interest in the role of n-3 LCPUFAs in cognitive function preservation, just one RCT evaluates the impact of n-3 LCPUFAs on age-related cognitive decline in post-menopausal women [75]. In this double-blind randomized controlled trial, 27 post-menopausal women were enrolled: the treated arm (*n* = 15) received a multinutrient supplement called Efalex Active 5, corresponding to 1 g DHA, 160 mg eicosapentaenoic acid, 240 mg *Ginkgo biloba*, 60 mg phosphatidyl-serine, 20 mg d-α tocopherol, 1 mg folic acid, and 20 μg vitamin B12, while the second arm (*n* = 12) received a placebo throughout a period of 24 weeks. Both groups were evaluated for mobility outcome measures, including Habitual walking (HW), Fast walking (FW), and Vertical jump height (VJH), and cognition outcome measures, including a battery of computer-based cognitive tests (CANTAB, Cambridge Cognition Ltd.), the psychomotor response latency “Motor Screening Task” (MOT), two memory tests, i.e., Verbal Recognition Memory (VRM), and Paired Associate Learning (PAL), and one executive function test (Stockings of Cambridge). The results of these assessments showed a significant improvement in two of the cognitive tests administered (MOT and VRM), and in one of the three primary mobility measures (HW speed).

The findings of this pilot study motivate further clinical trials on wider populations, possibly including women with cognitive impairment, to clarify the potential protective and therapeutic role of LCPUFAs in cognitive symptoms menopause-related. 

### 2.2. N-3 LCPUFAs in Menopausal Hot Flashes

Hot flashes (HFs) or VMS are the cardinal symptoms of menopause. HFs are mainly characterized by a subjective sensation of heat that may be associated with sweating, cutaneous vasodilatation, increased heart rate, and a subsequent drop in core temperature [81]. As far a possible biologic role, n-3 LCPUFAs seem to affect serotonergic transmission, similar to antidepressants, which have been demonstrated to diminish VMS [72]. However, the evidence that n-3 LCPUFAs may be efficacious for the treatment of hot flashes are controversial.

In this paragraph, we provide an update regarding the effects of n-3 LCPUFAs supplementation on VMS in menopausal women. We identified five RCT studies (Table 2).

A preliminary study by Freeman et al. (2011) evaluated the potential role of n-3 LCPUFAs for MDE and VMS during the menopausal transition [72]. They found a significant improvement in the mean frequency of hot flashes over a 24-h period with a significant decreased of Hot Flash Related Daily Interference Scale (HFRDIS) scores, i.e., a 10-item self-report questionnaire that measures the degree to which hot flashes interfere with daily activities and quality of life during the prior week. Furthermore, participants who were responders to treatment, evaluated based on the scores of depressive measures, were significantly more likely to have decreased hot flash diary scores than non-responders. Similarly, the RCT by Lucas et al. (2009) found positive results. Specifically, after eight weeks, HF frequency and scores decreased significantly in the Ethyl-Eicosapentaenoic (E-EPA) group compared with the placebo group. In particular, the number of daily HFs decreased by a mean of 1.58 (95% CI, −2.18 to −0.98), corresponding to 55% of the baseline value, in the E-EPA group and by 0.50 (95% CI, −1.20 to 0.20) (25% of baseline value) in the placebo group [71].

In contrast, a RCT study carried out by Cohen et al. (2014) testing the efficacy of omega-3s for the reduction in frequency and bother of VMS in peri- and postmenopausal women found that this supplementation did not significantly reduce hot flash frequency compared to placebo (*p* = 0.28) [73]. These results are reported also by Guthrie et al. (2015) in an analysis of pooled individual-level data from three RCTs (MsFLASH 01, MsFLASH 02, MsFLASH 03) [82]. Finally, Reed et al. (2014) confirmed that hot flash interference, stress, pain and sexual function showed no improvement with n-3 PUFAs interventions over usual care or placebo [83].

In conclusion, large high-quality RCTs are still required to further clarify the role of n-3 LCPUFAs on vasomotor symptoms in this population and to draw conclusions.

## 3. Materials and Methods

A comprehensive search on PUBMED of all trails using n-3 LCPUFAs on menopausal female patients with depressive and cognitive symptoms, and vasomotor symptoms published up to April 2018 was performed.

Articles of potential interest were identified by using the following search terms: “omega-3“, ”polyunsaturated fatty acids”, “long-chain polyunsaturated fatty acids”, “PUFAs”, “LCPUFAs” “EPA”, “DHA”, “docosahexaenoic acids”, “eicosapentaenoic acids” combined with the following term: “major depressive disorder”, “affective disorder”, “depression”, “depressive symptoms”, “hot flashes”, “vasomotor symptoms”, “cognitive disorder”, “cognitive symptoms”, “cognition” AND “menopausal transition”, “perimenopausal”, “postmenopausal”, “menopause”. In this review, trials examining the efficacy of n-3 LCPUFAs in menopausal women with depressive, cognitive, and vasomotor symptoms were selected.

Trials were included if they examined the efficacy of n-3 LCPUFAs to target depressive or cognitive disorders, and hot flashes in perimenopausal women.

We considered only trials in which the authors used an exposure of n-3 LCPUFAs as a unique treatment or as an adjunctive therapy to other drugs (e.g., hormone replacement therapy, antidepressants), or other no pharmacological strategies such as psychotherapy, physical exercise and phytoestrogens, compared to placebo or pharmacotherapy alone.

To limit the heterogeneity of this review and to reduce selection biases, we decided to exclude: trials examining the efficacy of n-3 LCPUFAs in subjects with others psychiatric diagnosis; trials analyzing levels of n-3 LCPUFA; studies that did not explore the effects of n-3 LCPUFAs on depressive or cognitive symptoms or hot flashes as primary outcome.

In addition, we excluded trials that employed a diet enriched in n-3 LCPUFAs as a supplementation.

Among the 238 articles retrieved, 16 studies were identified and screened by reading the abstract, and, when necessary, the full text, in order to select those articles relevant for the analysis. A manual search of bibliographic cross-referencing complemented the search. Reference lists of relevant papers were also inspected to identify any additional trials.

Relevant articles were obtained and included in the review if (a) they used an exposure of n-3 LCPUFAs; (b) included depressive, cognitive symptoms and hot flashes as an outcome measure and (c) enrolled human participants and reported a trial.

**Figure 1:**
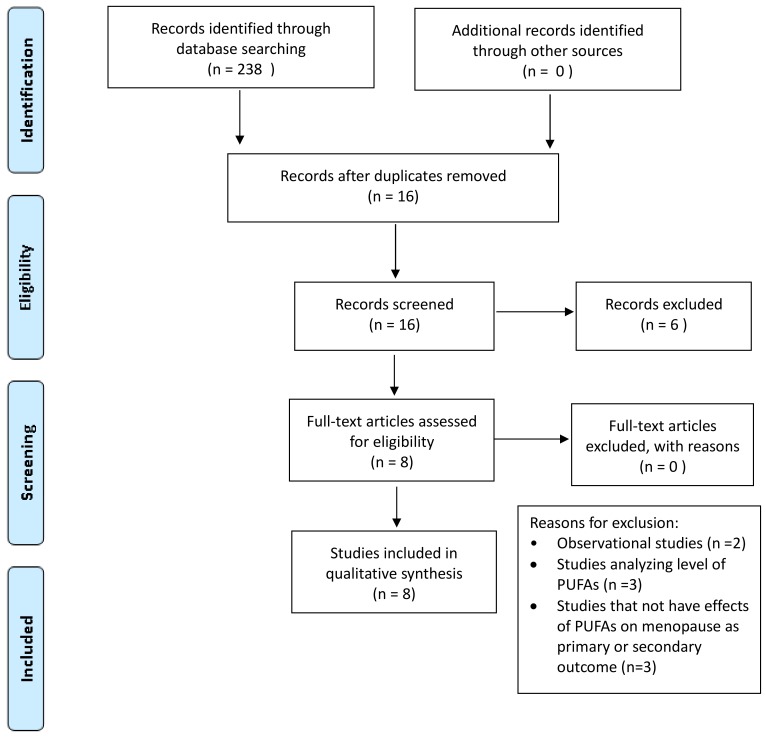
Preferred Reporting Items of Systematic reviews and Meta-Analyses (PRISMA) diagram: n-3 LCPUFAs supplementation in menopausal depressive and cognitive symptoms and hot flashes.

The process of identification and inclusion of trials is summarized in Figure 1. Finally, eight trials were included for the review. All searches, trial identification, data abstraction, and tabulation were completed independently by eight researchers. Discordances were discussed and resolved.

## 4. Conclusions and Future Direction

We have reviewed the effects of n-3 LCPUFAs in the treatment of hot flashes, and depressive and cognitive disorders linked to menopausal transition (Table 3). The interest for this alternative therapy raised from the evidence suggesting that the consumption of n-3 LCPUFAs has shown several beneficial effects on chronic-degenerative disorders, including cardiovascular, metabolic, neuropsychiatric, and inflammatory effects, which make it interesting for the women in menopausal transition.

Indeed, menopausal transition is accompanied by a number of symptoms of which hot flashes, depression, irritability, difficulties in concentrating are the most frequent. These symptoms are linked to the reduced production of estrogen causing changes in thermoregulation and in the levels of neurotransmitters, mainly serotonin. Our thorough overview of the literature has identified only a number of trends. Firstly, the few trials conducted on this topic reported that n-3 LCPUFAs, alone or added to other kind of pharmacological or no pharmacological interventions, might have the ability to alleviate menopausal symptoms [71,72,74,82]. Secondly the available evidences also suggest that the detection and treatment of n-3 LCPUFAs deficiency could be probably more effective in women that showed pretreatment lower n-3 LCPUFAs plasma levels [72]. However, this hypothesis requires future investigations. Thirdly, not all the available studies reported positive findings. Indeed, two trials reported no effect of n-3 LCPUFAs on hot flashes and depressive symptoms [73,83]. This might be probably due to the heterogeneity of the methods employed by the original studies, which often had small and not homogeneous sample size, different selection criteria, different subtypes and dosage of n-3 LCPUFAs (i.e., EPA, or DHA, or a combination of the two, or the addition of n-6 LCPUFAs as well as various duration of supplementation).

Another relevant methodological limitation is represented by the multitask nature of the neuropsychological tests and scales, leading to results not adjusted for multiple tests, which might therefore limit the generalizability of the findings. Finally, no adverse effects related to the treatment of n-3 LCPUFAs were observed in any of the clinical studies taken into account.

In conclusion, the lack of consistency across studies that have explored the n-3 LCPUFAs effects in perimenopausal disorders implies the necessity of larger prospective interventional clinical studies to elucidate the subtypes (EPA or DHA, or both types) and the therapeutic dose of n-3 LCPUFAs required in these kinds of perimenopausal disorders. On a methodological standpoint newer and more objective approaches should be used, either on an instrumental and a neurocognitive approach. Spectrometry-based studies may represent a way to directly follow the metabolic fate of a specific substrate towards a predicted function. These new approaches could help elucidate the wide-reaching implications of n-3 LCPUFAs in health and disease.

## Figures and Tables

**Table 1:** Randomized controlled trials (RCTs) of n-3 long-chain polyunsaturated fatty acids (n-3 LCPUFAs) on menopausal depressive and cognitive symptoms.

Depressive and Cognitive Symptoms	*n* Sample	N-PUFA Assessed Daily Amounts	Duration (Weeks)	Outcome Measures	Major Finding
Study					
[72]	19 postmenopausal women	Lovaza 2 g/day (1-g capsule: 465 mg EPA + 375 mg DHA + 160 mg small amounts of other omega-3 fatty acids)	8 weeks	The primary outcome was change in depressive symptoms from beginning to end of the study, as measured by MADRS	The depressive symptoms improved with treatment with a significant decrease in MADRS scores
[71]	120 post-menopausal women omega-3s (*n* = 59) or placebo (*n* = 61)	A 500-mg capsule three times daily (350 mg of EPA and 50 mg of DHA in the form of ethyl ester)	8 weeks	Primary outcome was to compare enriched (E-EPA) supplementation with placebo for the treatment of PD measured by (PGWB) and depressive symptoms measured by HSCL-D-20 and HAM-D-21	Supplementation with E-EPA omega-3 fatty acid improved significantly more than placebo in women with PD without MDE at baseline, but not significantly in women with PD and with MDE
[73]	355 post-menopausal women were randomly assigned to receive omega-3s (*n* = 177) or placebo (*n* = 178)	1.8 g/day of omega-3 supplementation (3 pills/day, each containing 425 mg of EPA, 100 mg DHA and 90 mg of other omega-3s)	12 weeks	The secondary outcomes were sleep quality measured by (PSQI), insomnia symptoms measured by (ISI), depressive symptoms measured by (PHQ-8), and anxiety measured by (GAD-7)	Omega-3s did not significantly reduce sleep or mood compared to placebo
[74]	60 postmenopausal women: *n* = 30 citalopram + 1 g omega-3s and *n* = 30 citalopram + placebo	1 g/day of omega-3 fish oil capsules	4 weeks	The effect of a combination of omega-3 and citalopram in the treatment of women with post-menopausal depression measured by BDI	Omega-3s in combination with citalopram demonstrated to reduce the severity of depression in post-menopausal wome
[75]	27 post-menopausal women: *n* = 15 received multinutrient supplement) and *n* = 12 received placebo	Four capsules per day of Efalex Active 50+, corresponding to: 1 g DHA, 160 mg eicosapentaenoic acid, 240 mg *Ginkgo biloba*, 60 mg phosphatidyl-serine, 20 mg d-α tocopherol, 1 mg folic acid, and 20 μg vitamin B12	24 weeks	The primary outcome measures were based on changes in mobility (including Habitual walking (HW), fast walking (FW), and Vertical jump height (VJH) and cognition (including psychomotor response latency (MOT), Verbal Recognition Memory (VRM), and paired associate learning (PAL))	Multinutrient supplement containing high doses of DHA and eicosapentaenoic acid significantly improves some cognition and mobility measures in post-menopausal women

DHA: Docosahexaenoic Acid; EPA: Eicosapentaenoic Acid; E-EPA: Ethyl-Eicosapentaenoic Acid; PD: Psychological Distress; MADRS: Montgomery-Asberg Depression Rating Scale; MDE : major depressive episode; PGWBS : Psychological General Well-Being Schedule; HSCL-D-20: 20-item Hopkins Symptom Checklist Depression Scale; HAM-D-21: 21-item Hamilton Depression Rating Scale:; PSQI :Pittsburg Sleep Quality Index; ISI: Insomnia Severity Index, PHQ-8: Physician’s Health Questionnaire-8; GAD-7: Generalized Anxiety Disorder-7; BDI: Beck’s Depression Inventory.

**Table 2:** RCTs of n-3 LCPUFAs in menopausal hot flashes.

Hot Flashes and Vasomotor Symptoms	*n* Sample	N-PUFA Assessed Daily Amounts	Duration (Weeks)	Outcome Measures	Major Finding
Study					
[72]	19 women	Lovaza 2 g/day (1 g capsule: 465 mg EPA + 375 mg DHA + 160 mg small amounts of other omega-3 fatty acids)	8 weeks	The secondary outcome was change in HF from beginning to end of the study, as measured by hot flash diary and HFRDIS scores	HF improved significantly with treatment, as evident in hot flash diary scores and HFRDIS scores
[71]	E-EPA, *n* = 43; placebo, *n* = 39	A 500-mg capsule three times daily (350 mg of EPA and 50 mg of DHA in the form of ethyl ester)	8 weeks	Secondary objectives were to compare the mean change in HFs (frequency, intensity, and score) and the proportion of HF responders (≥50% reduction in HF frequency between baseline and week 8)	Supplementation with E-EPA omega-3 fatty acid reduced HF frequency and improved the HF score relative to placebo
[73]	355 women were randomly assigned to receive omega-3s (*n* = 177) or placebo (*n* = 178)	1.8 g/day of omega-3 supplementation (3 pills/day, each containing 425 mg of EPA, 100 mg DHA and 90 mg of other omega-3s)	12 weeks	The primary outcomes were VMS frequency and bother based on daily diaries at baseline and weeks 6 and 12	Omega-3s did not significantly reduce hot flash frequency compared to placebo (*p* = 0.28)
[82]	177 women to omega-3 and 178 to placebo	1.8 g/day of omega-3 fish oil capsules (425 mg E-EPA acid, 100 mg DHA and 90 mg of other omega-3s three times a day)	12 weeks	The MsFLASH Network, has conducted three large RCTs for treatment of menopausal VMS testing six interventions including omega-3 fatty acid supplementation	The MsFLASH 02 interventions of yoga, exercise, and omega-3 showed little effect in reducing vasomotor symptom frequency or bother relative to control
[83]	355 women	1.8 g/day of omega-3 (425 mg E-EPA, 100 mg DHA and 90 mg of other omega-3s)	12 weeks	MENQOL total and domain (VMS, psychosocial, physical and sexual) scores	Hot flash interference, stress, pain and sexual function showed no improvement with exercise or omega-3 interventions over usual care or placebo, respectively

DHA: Docosahexaenoic Acid; EPA: Eicosapentaenoic Acid; E-EPA: Ethyl-Eicosapentaenoic Acid; HFRDIS: Hot Flash Related Daily Interference Scale scores; HF: hot flashes; VMS: vasomotor symptoms; MENQOL: Menopausal Quality of Life Questionnaire.

**Table 3:** Effects of n-3 LCPUFAs supplementation in menopausal depressive and cognitive symptoms and hot flashes: summary.

Menopausal Symptoms	Positive Results	Negative Results	Positive Results without Statistical Significance
Hot flashes	[71,82]	[73,83]	[72]
Depressive symptoms	[74]	[73]	[71,72]
Cognitive symptoms	[75]		
